# Host-Induced Genome Instability Rapidly Generates Phenotypic Variation across Candida albicans Strains and Ploidy States

**DOI:** 10.1128/mSphere.00433-20

**Published:** 2020-06-03

**Authors:** Amanda C. Smith, Meleah A. Hickman

**Affiliations:** a Department of Biology, Emory University, Atlanta, Georgia, USA; University of Georgia

**Keywords:** *Candida albicans*, fitness, genome stability, host-pathogen interactions

## Abstract

Candida albicans is an opportunistic fungal pathogen of humans that is typically diploid yet has a highly labile genome tolerant of large-scale perturbations including chromosomal aneuploidy and loss-of-heterozygosity events. The ability to rapidly generate genetic variation is crucial for C. albicans to adapt to changing or stressful environments, like those encountered in the host. Genetic variation occurs via stress-induced mutagenesis or can be generated through its parasexual cycle, in which tetraploids arise via diploid mating or stress-induced mitotic defects and undergo nonmeiotic ploidy reduction. However, it remains largely unknown how genetic background contributes to C. albicans genome instability *in vitro* or in the host environment. Here, we tested how genetic background, ploidy, and the host environment impacts C. albicans genome stability. We found that host association induced both loss-of-heterozygosity events and genome size changes, regardless of genetic background or ploidy. However, the magnitude and types of genome changes varied across C. albicans strain background and ploidy state. We then assessed if host-induced genomic changes resulted in fitness consequences on growth rate and nonlethal virulence phenotypes and found that many host-derived isolates significantly changed relative to their parental strain. Interestingly, diploid host-associated C. albicans predominantly decreased host reproductive fitness, whereas tetraploid host-associated C. albicans increased host reproductive fitness. Together, these results are important for understanding how host-induced genomic changes in C. albicans alter its relationship with the host.

**IMPORTANCE** Candida albicans is an opportunistic fungal pathogen of humans. The ability to generate genetic variation is essential for adaptation and is a strategy that C. albicans and other fungal pathogens use to change their genome size. Stressful environments, including the host, induce C. albicans genome instability. Here, we investigated how C. albicans genetic background and ploidy state impact genome instability, both *in vitro* and in a host environment. We show that the host environment induces genome instability, but the magnitude depends on C. albicans genetic background. Furthermore, we show that tetraploid C. albicans is highly unstable in host environments and rapidly reduces in genome size. These reductions in genome size often resulted in reduced virulence. In contrast, diploid C. albicans displayed modest host-induced genome size changes, yet these frequently resulted in increased virulence. Such studies are essential for understanding how opportunistic pathogens respond and potentially adapt to the host environment.

## Abstract (precis)

Candida albicans is an opportunistic fungal pathogen of humans. The ability to generate genetic variation is essential for adaptation and is a strategy that C. albicans and other fungal pathogens use to change their genome size. Stressful environments, including the host, induce C. albicans genome instability. Here, we investigated how C. albicans genetic background and ploidy state impact genome instability, both *in vitro* and in a host environment. We show that the host environment induces genome instability, but the magnitude depends on C. albicans genetic background. Furthermore, we show that tetraploid C. albicans is highly unstable in host environments and rapidly reduces in genome size. These reductions in genome size often resulted in reduced virulence. In contrast, diploid C. albicans displayed modest host-induced genome size changes, yet these frequently resulted in increased virulence. Such studies are essential for understanding how opportunistic pathogens respond and potentially adapt to the host environment.

## INTRODUCTION

Host-pathogen interactions are multifaceted. As a fungal opportunistic pathogen of humans, Candida albicans has many different relationships with the host. Typically, C. albicans is commensal, residing in many niches in the human body, including the gastrointestinal and urogenital tracts, oral cavity, and skin (1). However, C. albicans can be pathogenic and cause superficial mucosal infections and deadly bloodstream infections (2, 3). Much of the research regarding C. albicans has focused on its virulence factors, which include filamentation, biofilm formation, secretory aspartyl proteinases (SAPs), and candidalysin production (4, 5). Host immune cells control C. albicans infection by recognizing fungal cells and producing antimicrobial peptides (AMPs) (6) and reactive oxygen species (ROS) which inhibit growth and cause DNA damage (7, 8). However, how host-induced genome alterations in C. albicans impact its relationship with the host is not well understood.

Genomic alterations in C. albicans have important consequences in other clinical contexts, including the acquisition of resistance to the drugs used to treat fungal infections. Analysis of clinical isolates and laboratory studies show that chromosomal aneuploidy and homozygosis of hyperactive resistance alleles are associated with increased antifungal drug resistance (9–14). The ability to generate genetic variation and tolerate genomic perturbations is a strategy that C. albicans leverages to adapt to changing environments (15). As a highly heterozygous diploid (16), the C. albicans genome is highly labile and undergoes large-scale genome rearrangements like loss-of-heterozygosity (LOH) events and aneuploidy more frequently than small-scale DNA mutations. Furthermore, exposure to physiologically relevant stress conditions, including high temperature, oxidative stress, and antifungal drugs, increases LOH rates even further (17, 18).

Another mechanism that C. albicans uses to generate genetic variation is parasex, which involves diploid-diploid mating to produce tetraploids (19, 20). Tetraploids undergo stochastic chromosome loss to return to diploid. This process is termed concerted chromosome loss and results in reassortment of alleles, loss of heterozygosity (LOH), and aneuploidy (21–23). The tetraploid LOH rate is substantially higher than in diploids (23). In the context of antifungal drugs, large-scale mutation rates are disproportionately elevated in tetraploids compared to diploids (18). Tetraploids, which undergo more genomic changes than diploids, have high potential to produce phenotypic changes with fitness consequences (23, 24). In fact, diploids will produce transient tetraploids via mitotic errors when exposed to high doses of the antifungal drug fluconazole (25). Therefore, tetraploidy may be a useful evolutionary strategy to produce genetic variation.

Clinical C. albicans tetraploids have been isolated from human hosts (24, 26–28), although whether they arise via parasex or stress-induced mitotic defects is not known. Furthermore, clinical diploid isolates also carry genomic changes such as aneuploidy and LOH (29). A small number of experimental studies have investigated C. albicans genome stability within host environments and found that host association elevates mutation rates over those *in vitro* (30–32). Host-induced genetic variation increases phenotypic variation, including colony morphology, hyphal formation, and virulence (30, 32–34). For example, chromosome 6 and chromosome 7 trisomies arise in C. albicans associated with murine hosts and display attenuated virulence (31, 33). While many studies use murine models to measure virulence (reviewed in reference 35), we previously developed a novel Caenorhabditis elegans infection model. Our host-pathogen system can assess not only host survival but also host reproduction (36), an often-overlooked aspect of virulence (37). Host fecundity offers a nonlethal phenotype, which can be important when studying virulence in opportunistic pathogens that cause a wide range of infections (37).

Here, we investigated how host association impacts C. albicans genome instability across multiple genetic backgrounds and ploidies. We used three diploid-tetraploid pairs of C. albicans strains from distinct genetic backgrounds to infect C. elegans hosts and subsequently measured LOH frequency and genome size changes in C. albicans. We found that host association increased genome instability for all C. albicans strains, but the degree to which it was elevated depended on strain background. Furthermore, host-associated diploids had minor but significant genome size changes, whereas host-associated tetraploids rapidly underwent major reductions in genome size. We assessed how these genomic changes altered strain fitness and virulence. Most diploid isolates were more virulent following host association, but many tetraploid isolates did not change virulence or were less virulent, despite undergoing massive genome size changes. Taken together, our results show that host association induced genetic variation in diploid and tetraploid C. albicans of diverse genetic backgrounds which impacted virulence phenotypes.

## RESULTS

### Host association elevates LOH events in laboratory and clinical C. albicans isolates.

**FIG 1:**
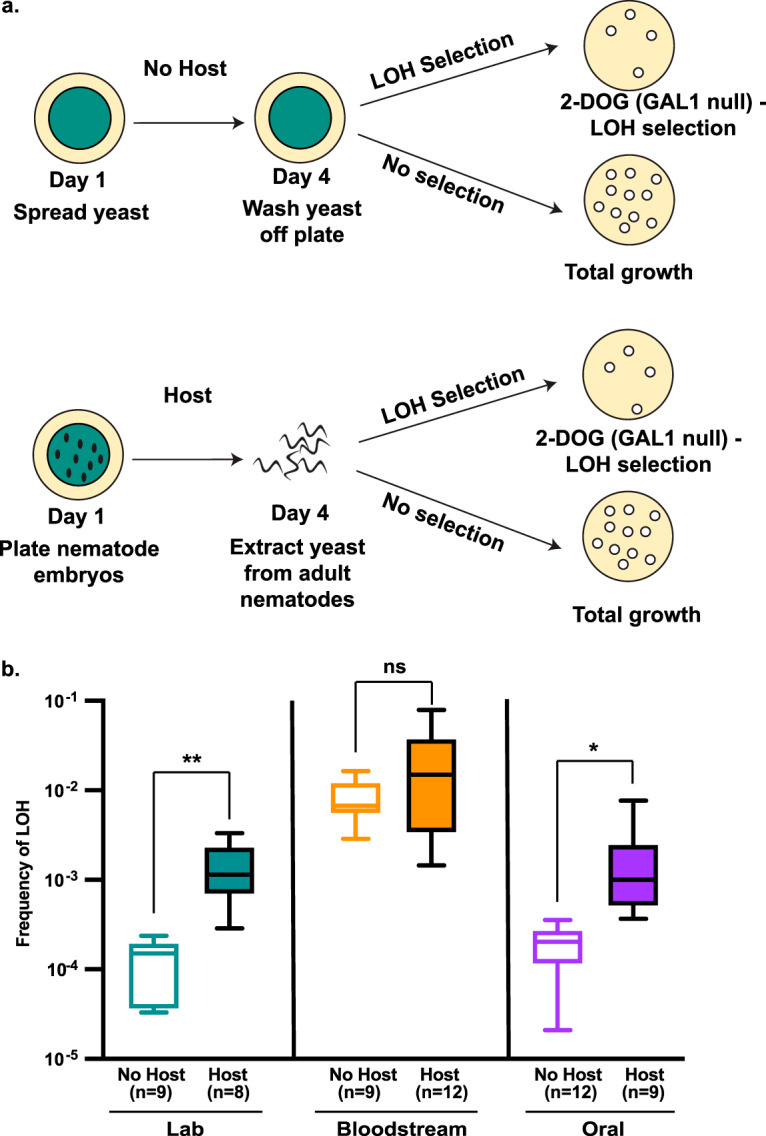
Host association induces LOH. (a) Experimental schematic. C. albicans was plated in either the presence or absence of hosts, subsequently extracted or washed from the treatment plates, and plated on medium that selected for LOH events (2-DOG) or rich medium (YPD) to determine total viable growth. (b) No-host and host-association *GAL1* LOH frequencies for laboratory (green), bloodstream (orange), and oral (purple) diploid C. albicans strains. Boxes represent the 25th and 75th quartile with the whiskers representing the total data range. Asterisks indicate significant differences between the no-host and host treatment groups for each strain background (*, *P* < 0.05; **, *P* < 0.01; ns, not significant; unpaired Student’s *t* test).

The laboratory strain of C. albicans shows elevated genome instability in murine host environments compared to *in vitro* (30). Here, we wanted to determine how C. albicans genetic background impacted *in vitro* and host-associated genome instability. Using a C. elegans infection model, we compared the frequency of loss of heterozygosity (LOH) at the *GAL1* locus (17) of a laboratory and two clinical diploid strains (Table 1) *in vivo* to the *in vitro* LOH frequency (Fig. 1a). If the host environment elevated C. albicans genome instability, then the host-associated LOH frequency should increase relative to that *in vitro*. For the laboratory and the oral clinical strains, we found that host-associated LOH is approximately 10-fold higher than that *in vitro* (Fig. 1b, teal and purple). However, the bloodstream clinical strain had no significant difference in LOH frequency between the *in vitro* and host-associated treatments, likely due to the high *in vitro* LOH frequency (Fig. 1b, orange). Indeed, we found significant differences among the strain backgrounds for the *in vitro* (*P* < 0.0001, Kruskal-Wallis test) and host-associated (*P* = 0.0008, Kruskal-Wallis test) treatments, with the bloodstream strain displaying higher instability than the other two strains (see Table S2 in the supplemental material). Our results are consistent with data from murine models (30–33) which support the hypothesis that host environments increase C. albicans genome instability and demonstrate that C. albicans genetic background influences genome instability.

**TABLE 1:** Strains used in this study

Strain	Type	Ploidy	Genetic background	Reference(s)
YJB11700^*a*^	Laboratory	Diploid (euploid 2N)	SC5314 reference strain; *GAL1/gal1*Δ::*NAT*	54, 55
RBY18	Laboratory	Tetraploid (euploid 4N)	Mating product between two SC5314-derived strains	21
FH1^*a*^	Bloodstream	Diploid (euploid 2N)	Initial clinical isolate from marrow transplant patient (same as FH6); *GAL1/gal1*Δ::*NAT*	27, 28
FH6^*a*^	Bloodstream	∼Tetraploid: 4xChr1, Chr2, Chr3, Chr7, ChrR; 3xChr4, Chr5, Chr6; 2x isochromosome (5L)	Midinfection, post-antifungal treatment, clinical isolate recovered from marrow transplant patient (same as FH1)	27, 28
PN2	Oral	∼Diploid: 2xChr1, Chr2, Chr3, Chr4, Chr5, Chr7, ChrR; 3xChr6	Clinical isolate recovered from the oral cavity from the same patient as PN1; *GAL1/gal1*Δ::*NAT*	24
PN1	Vaginal	Tetraploid (euploid 4N)	Clinical isolate recovered from vaginal infection post-antifungal treatment from the same patient as PN2	24

a Strains with genome sequences available.

### Host association induces genome size changes in clinical diploid strains.

**FIG 2:**
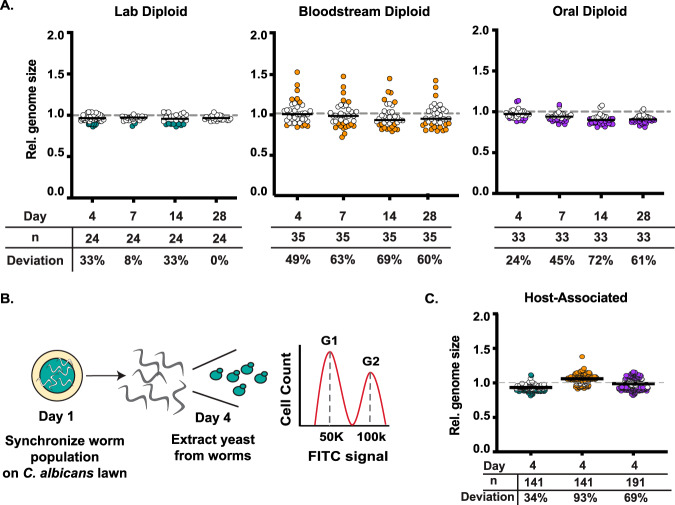
Diploid genome size stability *in vitro* and *in vivo.* (A) Relative genome size of laboratory, bloodstream, and oral diploid replicate lines passaged for 28 days in rich medium. Symbols represent individual replicate lines, and the median is indicated by the solid black line. Filled symbols indicate replicate lines more than 1 standard deviation away from the day 0 mean and reflect deviations from the initial diploid state. (B) Experimental schematic for assessing host-associated genome changes. (C) Relative genome size of laboratory, bloodstream, and oral host-associated isolates. Isolates were extracted from the gut, and genome size was measured via flow cytometry. Symbols represent individual C. albicans colonies extracted from the host, and the median is indicated by the solid black line. Filled symbols indicate replicate lines more than 1 standard deviation away from the no-host mean and reflect deviations from the initial diploid state.

Since strain background impacts LOH frequency (Fig. 1), we next wanted to examine if it also affects genome size changes over time. We have previously shown that clinical strains are more unstable than the laboratory strain (24); however, we did not investigate changes over the course of serial passaging. To identify *in vitro* genome size changes over time, we passaged 60 replicate lines for each genetic background in nutrient-rich liquid medium for 28 days and periodically measured genome size via flow cytometry for every replicate line. To assess whether replicate lines deviated in genome size changes over time, we plotted the genome size for each replicate population as the fraction of its initial genome size. Most replicate lines maintained their initial genome size throughout the 28-day experiment, regardless of strain background. However, the number of replicate lines that deviated from diploidy was higher for the clinical genetic backgrounds than for the laboratory strain (Fig. 2A, “deviation”). For example, on day 14, the clinical bloodstream and oral strains had 69% and 72% of their replicate lines with deviations from their initial diploid state, respectively. In contrast, only 33% of laboratory replicate lines deviated from diploidy by that same time point. Remarkably, the bloodstream strain had deviations with both major gains (i.e., 1.5×) and losses (i.e., 0.5×) in genome size that suggest that haploidy and triploidy can arise in this strain background (Fig. 2A and Fig. S1A). For the oral strain, the majority of the deviations in diploidy were minor losses in genome size (Fig. S1A). In addition to minor losses in genome size, in the laboratory strain, multiple G_1_ peaks were observed during flow cytometry for several replicate lines on day 4, indicating mixed populations. However, by day 28, all of the laboratory replicate lines resolved back to a diploid state (Fig. 2A and Fig. S1A). Together, these data demonstrate that the two clinical strains are more likely to generate genome size changes than the laboratory strain during *in vitro* passaging.

10.1128/mSphere.00433-20.1 FIG S1 Types of genomic changes observed in diploids and tetraploids. (A) The percentage of genome size gains and losses in diploid strains. Flow cytometry was used to determine genome size. Gains in genome size (light blue) represent replicate lines greater than 1 SD from the day 0 mean, and losses in genome size (dark blue) are less than 1 SD from the day 0 mean. Replicate lines with more than one G_1_ flow cytometry peak were considered mixed populations (green and white). (B) Same analysis as in panel A but for host-associated diploids. (C) Same analysis as in panel A but for each of the tetraploids *in vitro*. (D) Same analysis as in panel A but for host-associated tetraploids. Download FIG S1, PDF file, 0.4 MB. Copyright © 2020 Smith and Hickman. 2020 Smith and Hickman This content is distributed under the terms of the Creative Commons Attribution 4.0 International license.

Next, we wanted to assess how the host impacts genome size stability for each of the three strains. Similarly to the LOH assay, we exposed hosts to C. albicans for 4 days and subsequently extracted the yeast on day 4. Colonies were picked at random, and flow cytometry was performed to assess genome size (Fig. 2B). As an *in vitro* control, we plated an equivalent lawn of C. elegans in the absence of nematode hosts, and we calculated the relative genome size for the host-associated isolates as a fraction of the genome size of the *in vitro* control not exposed to the host. The laboratory strain had the lowest number of isolates that deviated from diploid (Fig. 2C, teal). Of the 34% of isolates that were no longer diploid, all had minor losses in genome size (Fig. 2C and Fig. S1B). In the oral diploid strain, 69% of host-associated isolates were no longer diploid. The deviations from diploid in this strain were both minor gains and losses (Fig. 2C, purple, and Fig. S1B). Shockingly, 93% of the bloodstream host-associated isolates were no longer diploid, after exposure to the host for 4 days. The majority of deviations from diploidy in this strain background were both major and minor gains in genome size (Fig. 2C, orange, and Fig. S1B). These results are consistent with the laboratory strain being more stable than the clinical strains, both *in vitro* and *in vivo*. Furthermore, our results indicate that the host environment increases genome instability, in terms of both LOH and genome size changes, yet the amount and direction of these genomic changes depend on strain background.

### Tetraploids undergo rapid genome size reduction in the host environment.

**FIG 3:**
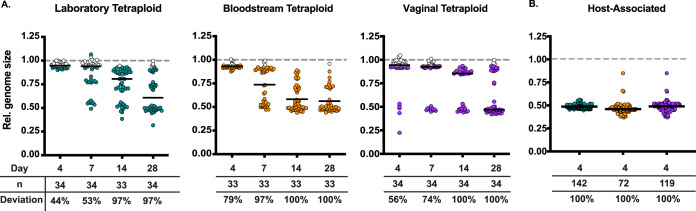
Tetraploid genome size stability *in vitro* and *in vivo*. (A) Relative genome size of laboratory, bloodstream, and vaginal tetraploid replicate lines passaged for 28 days in rich medium. Symbols represent individual replicate lines, and the median is indicated by the solid black line. Filled symbols indicate replicate lines more than 1 standard deviation away from the day 0 mean and reflect deviations from the initial tetraploid state. (B) Relative genome size of laboratory, bloodstream, and vaginal host-associated isolates. Isolates were extracted from the gut, and genome size was measured via flow cytometry. Symbols represent individual C. albicans colonies extracted from the host, and the median is indicated by the solid black line. Filled symbols indicate replicate lines more than 1 standard deviation away from the no-host mean and reflect deviations from the initial tetraploid state.

Our previous work demonstrated that tetraploid strains do not maintain tetraploidy during long-term passaging (23, 24). However, the dynamics of tetraploid instability for clinical strains was not captured. Therefore, we wanted to analyze the genome size stability of diverse tetraploid strains over time. We used three tetraploid strains: the laboratory tetraploid strain, a mating product between two laboratory diploids (21); a bloodstream clinical tetraploid recovered from a marrow transplant patient (27, 28); and a vaginal clinical strain recovered from a vaginal infection (24). To measure genome size changes over time *in vitro*, we passaged 60 replicate lines for each tetraploid genetic background in nutrient-rich liquid medium for 28 days and periodically measured genome size via flow cytometry for each replicate line. To assess whether replicate lines deviated in genome size changes over time, we plotted the genome size for each replicate population as the fraction of its initial genome size. Unlike diploids, most tetraploid replicate lines did not maintain their initial genome size; instead, most underwent genome reduction to about half their initial genome content (i.e., approximately diploid after passaging), regardless of strain background. By day 28, 100% of the vaginal and bloodstream replicate lines were no longer tetraploid, and 97% of laboratory replicate lines were not tetraploid (Fig. 3A and Fig. S1C). However, we detected genome size changes at earlier time points for the clinical strains than the laboratory strain. By day 7, more clinical replicate lines (97% and 74% for bloodstream and vaginal isolates, respectively) had a reduction in genome size compared to the laboratory strain, in which only 53% of replicate lines had a reduction in genome size. Similarly to our previous work (24), we demonstrated that tetraploids reduce to diploidy after serial passaging. Additionally, we demonstrated that the clinical tetraploids undergo a more rapid reduction in genome size than the laboratory strain during *in vitro* passaging.

Since the host environment induces genome size changes in C. albicans diploid strains (Fig. 1) (26–28), we next wanted to assess how the host impacts tetraploids. To assess host-associated tetraploid stability, we infected a population of C. elegans with each one of the three tetraploid strains mentioned above. After 4 days of exposure to C. albicans, we extracted C. albicans from the C. elegans gut via manual grinding and subsequently measured the genome size of the host-associated isolates via flow cytometry. We then calculated the relative genome size compared to the *in vitro* controls for each strain background. After only 4 days of host association, 100% of all host-associated single-colony isolates were no longer tetraploid, regardless of strain background. Furthermore, the vast majority of these tetraploid-derived isolates had approximately one-half of their initial genome content and are likely diploid (Fig. 3B). While we anticipated elevated tetraploid instability associated with the host environment, this result was surprising, given the short time frame of host association and the strain-independent nature. When we compare the host-associated results to the *in vitro* 28-day passaging, we observed that some replicate lines could maintain tetraploidy *in vitro* but not *in vivo*. Furthermore, while the clinical tetraploid strains showed more genome size variation than the laboratory strain during *in vitro* passaging, these size changes were more modest than the host-associated genome size changes. Together, these data suggest that the host environment rapidly reduces the genome size of tetraploids.

### Host-induced genome size changes impact C. albicans growth and virulence.

**FIG 4:**
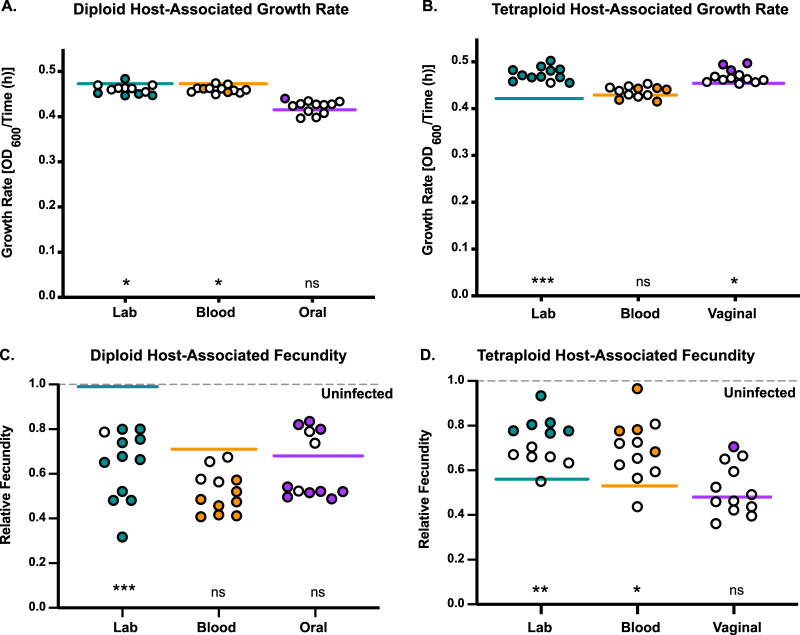
Growth rate and virulence phenotypes associated with host-induced genomic changes. (A) Growth rate of the laboratory (green), bloodstream (orange), and oral (purple) host-associated isolates. Each solid line represents the mean growth rate of the parental strain. The filled-in circles represent host-associated growth rates that are significantly different from the mean growth rate of the parental strain (Mann-Whitney U). (B) Same analysis as in panel A but with tetraploid host-associated isolates. (C) Relative fecundity of host infected with laboratory (green), bloodstream (orange), and oral (purple) host-associated isolates. Relative fecundity was calculated by dividing the total brood size of infected hosts by the total brood size of uninfected hosts. Solid lines represent the mean relative fecundity for each parental strain. Filled-in circles are significantly different from the parental strain; open circles are not (Mann-Whitney U). (D) Same analysis as in panel C but with tetraploid host-associated isolates. We tested for differences between the host-associated isolates and their respective parents using a Mann-Whitney U test (***, *P* < 0.0001; **, *P* < 0.01; *, *P* < 0.05; ns, not significant).

Since host association induced genomic changes in C. albicans, we next wanted to assess if these changes had any fitness consequences for growth rate. We focused on host-associated derivatives with genome size changes, since genome size changes were not biased by LOH marker selection, and we did not have host-associated LOH isolates from our tetraploid strains. For each strain, we selected 12 host-associated isolates (Table S3), measured their growth rate in nutrient-rich medium, and compared that to the growth rate of their respective parental strain (Fig. 4A and B). It is important to note that there were some differences between genetic background and ploidy state in the parental strains. For each genetic background, the diploid growth rate was significantly different from its corresponding tetraploid growth rate (Table 2). For the laboratory and bloodstream ploidy pairs, the tetraploid grew more slowly than the diploid, but for the oral/vaginal genetic background, the opposite was observed. Furthermore, within each ploidy class, the laboratory and bloodstream strains were significantly different from the oral/vaginal strains (Table 2). When we compared the host-associated isolates to their respective parental strains, we found collectively that derivatives from all strain backgrounds except the oral diploid and bloodstream tetraploid were significantly different (Fig. 4A and B). Furthermore, the diploid host derivatives had decreased growth rates and the tetraploid genetic backgrounds had increased growth rates. When we compared the host-derived isolates as individuals to their parental strains, we detected some minor yet significant changes in growth for all strain backgrounds and ploidies (Fig. 4A and B, filled symbols).

**TABLE 2:** Parental strain growth rates^*a*^

Strain	Diploid			Tetraploid		
	Laboratory	Bloodstream	Oral	Laboratory	Bloodstream	Vaginal
Mean growth rate	0.47	0.47	0.42	0.42	0.44	0.45
SD	0.02	0.01	0.01	0.02	0.01	0.03
*n*	12	12	12	12	12	12

Diploid						
Laboratory						
Bloodstream	NS					
Oral	****	****				

Tetraploid						
Laboratory	****	****	NS			
Bloodstream	****	****	NS	NS		
Vaginal	*	*	**	*	**	

a Mean growth rate, standard deviation (SD), and number of biological replicates (*n*) are indicated for each treatment. Differences between treatments were tested in all pairwise combinations using the Mann-Whitney U test, and significance is indicated (NS, not significant; *, *P* < 0.05; **, *P* < 0.01; ***, *P* < 0.001; ****, *P* < 0.0001).

Decreases in virulence or changes to commensal-like phenotypes have been previously shown following short- and long-term association with host environments (33, 34). We have previously shown that C. albicans virulence can be assessed in C. elegans as lethal (i.e., host survival) and nonlethal (i.e., fecundity) phenotypes that reduce host fitness (36). To assess whether host-induced genome size changes altered virulence phenotypes, we measured the reproductive success (i.e., fecundity) of C. elegans hosts infected individually with each parental strain and their 12 host-derived isolates and calculated the relative fecundity by dividing the brood size of infected hosts by the brood size of uninfected hosts (Fig. 4C and D). It should be noted that there were some differences between genetic background and ploidy state in the parental strains. The diploid-infected host fecundity was significantly higher than its corresponding tetraploid-infected host fecundity for the laboratory and oral/vaginal genetic backgrounds, indicating that tetraploids are generally more virulent than diploids (Table 3). We also found that genetic background impacted host fecundity for diploids but not tetraploids (Table 3).

**TABLE 3:** Relative fecundity of parental strain and infected host^*a*^

Strain	Diploid			Tetraploid		
	Laboratory	Bloodstream	Oral	Laboratory	Bloodstream	Vaginal
Mean relative fecundity	0.91	0.71	0.68	0.56	0.52	0.48
SD	0.11	0.1	0.14	0.1	0.12	0.13
*n*	27	15	18	10	6	7

Diploid						
Laboratory						
Bloodstream	*					
Oral	****	NS				

Tetraploid						
Laboratory	****	*	*			
Bloodstream	****	NS	*	NS		
Vaginal	****	*	*	NS	NS	

a Relative fecundity (infected brood size/uninfected brood size), standard deviation (SD), and number of biological replicates (*n*) are indicated for each treatment. Differences between treatments were tested in all pairwise combinations using the Mann-Whitney U test, and significance is indicated (NS, not significant; *, *P* < 0.05; **, *P* < 0.01; ***, *P* < 0.001; ****, *P* < 0.0001).

When we compared the host-associated isolates to their respective parental strains, we found collectively that derivatives from the laboratory diploid and tetraploid and the bloodstream tetraploid were significantly different (Fig. 4C and D). When we compared the host-derived isolates as individuals to their respective parental strains, there were significant changes to host fecundity for all strain backgrounds and ploidies (Fig. 4C and D, filled symbols). In general, many of the diploid host-derived isolates were more virulent (i.e., reduced brood sizes) than their respective parental strains, with the exception of a small number of the host-derived isolates of the vaginal strain background that displayed reduced virulence. All of the tetraploid host-associated isolates that significantly differed showed reductions in virulence (i.e., larger brood sizes). However, there was no direct correlation between genome size changes and relative fecundity or growth (Fig. S2). Taken together, our results indicate that even short periods of host association induce genomic changes that have direct impacts on virulence phenotypes.

10.1128/mSphere.00433-20.2 FIG S2 Correlation between genome size changes and fitness or virulence in both tetraploid and diploid C. albicans host-associated isolates. (A) Relationship between relative growth rate and relative genome size for host-associated laboratory (green), bloodstream (orange), and oral (purple) diploid isolates. Pearson correlation: laboratory, *r* = −0.08, *P* = 0.805; bloodstream, *r* = 0.18, *P* = 0.57; oral, *r* = 0.38, *P* = 0.22. (B) Relationship between the relative fecundity and the relative genome size for host-associated laboratory (green), bloodstream (orange), and oral (purple) diploid isolates. Pearson correlation: laboratory, *r* = −0.57, *P* = 0.051; bloodstream, *r* = −0.31, *P* = 0.33; oral, *r* = 0.42, *P* = 0.18. (C) Relationship between relative growth rate and relative genome size for host-associated laboratory (green), bloodstream (orange), and oral (purple) tetraploid isolates. Pearson correlation: laboratory, *r* = −0.24, *P* = 0.45; bloodstream, *r* = 0.08, *P* = 0.81; vaginal, *r* = −0.23, *P* = 0.47. (D) Relationship between the relative fecundity and the relative genome size for host-associated laboratory (green), bloodstream (orange), and oral (purple) tetraploid isolates. Pearson correlation: laboratory, *r* = −0.09, *P* = 0.78; bloodstream, *r* = −0.04, *P* = 0.91; vaginal, *r* = −0.33, *P* = 0.29. Download FIG S2, PDF file, 0.4 MB. Copyright © 2020 Smith and Hickman. 2020 Smith and Hickman This content is distributed under the terms of the Creative Commons Attribution 4.0 International license.

## DISCUSSION

Here, we investigated how diverse genetic backgrounds and ploidy states of C. albicans impact genome stability both inside and outside the host environment. Previous work has shown that differences in genetic background give rise to phenotypic differences (29) and also impact long-term genome dynamics (24). Furthermore, earlier studies have shown that the host environment increases genome instability (30–33), but these studies do not address the roles that genetic background and ploidy play in host-associated genome dynamics. By using three diploid-tetraploid pairs of C. albicans strains with distinct genetic backgrounds, we were able to compare strain differences in genome stability both *in vitro* and *in vivo*. Furthermore, we analyzed how host-induced genetic changes impacted C. albicans growth and nonlethal host fitness phenotypes. We found that host-association increased genome instability relative to *in vitro* for all strain backgrounds (Fig. 1). However, the magnitude by which the host elevated genome instability was dependent on strain background. We observed that diploids had minor genome size changes in the host environment (Fig. 2), whereas our three tetraploid strains underwent rapid and large genome reductions in the host environment (Fig. 3). Finally, when assessing whether host-induced genome size changes impacted host reproductive fitness, we found that diploid derivatives generally increased in virulence and tetraploids generally decreased in virulence (Fig. 4).

We were surprised to detect significant virulence changes in isolates derived from diploid-infected hosts, since we detected only modest genome size changes relative to tetraploids. However, 28 out of the 36 host-associated isolates had significant changes in virulence compared to their parental strain (Fig. 4C). Host reproduction generally decreased when the host was infected with diploid derivatives. Interestingly, 25% (3 of 12) diploid derivatives from the oral strain increased host brood sizes, indicating reduced virulence. Genetic background was also important for diploid genome stability. For example, host-induced LOH was ∼10-fold elevated in the laboratory and oral strain backgrounds but only 2-fold elevated in the bloodstream strain. This is most likely due to the very high *in vitro* LOH frequency of the bloodstream strain. Our findings are consistent with whole-genome sequencing studies that show naturally occurring LOH events in clinical isolates (29, 38–40). These sequencing studies also detected chromosomal aneuploidy in a small number of clinical strains (28, 29, 39). In our work, we observed that clinical diploid strains undergo minor genome size changes more frequently over the course of *in vitro* passaging and host association compared to the laboratory strain (Fig. 2). This study builds upon previous experimental studies which found that host environments elevate genome instability in C. albicans (30–32), and we extend this by explicitly testing for differences in multiple genetic backgrounds as well as in tetraploids.

Tetraploids underwent massive genome reductions when exposed to the host environment regardless of genetic background, and in contrast to diploids, which had significant but modest genome size changes. We propose that the host environment is inherently stressful and drives genome instability in C. albicans similarly to stress-induced mutagenesis. There are several physiologically relevant stressors that elevate LOH rates *in vitro*, including high temperature and oxidative stress (17, 41). Reactive oxygen species (ROS) production is an innate immune defense used to defend the host against invading pathogens (7, 8), which inhibits growth by inducing DNA damage (42). Our results indicate that host-induced genome instability could result from host ROS production. Given that C. elegans has a conserved innate immune system that includes producing ROS to defend against pathogens (43–45), it would be interesting to investigate how immune function contributes to pathogen genome instability.

The extreme genome instability observed in tetraploids is partly due to their intrinsic highly labile nature. It has been well established that tetraploid C. albicans has higher levels of genome instability than diploids (18, 22–24), and this phenomenon is also observed in related yeast species (46–49). However, knowledge of how genetic background impacts tetraploid genome stability has been extremely limited to date (24). We have previously shown strain-dependent differences in tetraploids following long-term *in vitro* serial passaging (24). In the current work, we detected early differences across genetic backgrounds in tetraploid genome reduction during *in vitro* serial passaging (Fig. 3A). From our collective *in vitro* results, we anticipated that host association would likely induce genome size changes to some degree in tetraploids. However, we were surprised to observe all tetraploid host derivatives with genome reductions, most of which were close to diploid in content (Fig. 3B), after only 4 days of host association. While tetraploid C. albicans organisms have been isolated in clinical settings (26–28), they are rare in comparison to diploid clinical isolates. Our results showing rapid host-induced tetraploid genome reduction may help explain the rarity of clinical tetraploids.

Host-induced genome changes resulted in subsequent changes in nonlethal virulence phenotypes for all strains (Fig. 4C and D). We did not anticipate much phenotypic variation because host-induced genetic changes arose in the absence of selection, so it was striking that ∼78% and ∼31% of diploid and tetraploid host-associated derivatives changed virulence relative to their parental strains, respectively. Not only were virulence changes more frequent in the diploid derivatives, but nearly all were increases in virulence (i.e., decreased host reproduction). In contrast, the few significantly different tetraploid host derivatives had reductions in virulence. Differences in baseline virulence between the parental diploid and tetraploid strains (Table 3) may partially explain this result, since tetraploids were generally more virulent than diploids. It should be noted that baseline differences in virulence between diploids and tetraploids have been observed previously, but in the opposite direction of our findings (50). However, these differences may stem from our choice of model system (nematode versus mouse) and virulence phenotype (lethal versus nonlethal).

Finally, we were surprised that so few of the host-derived tetraploids changed in virulence, given the frequency and magnitude of genome size changes induced in the host environment. There was no correlation between changes in genome size and changes in virulence (Fig. S2), similar to previous work demonstrating that *in vitro* adaptation to nutrient depletion is not correlated with genome size changes (24). Tetraploid genome reduction generates massive karyotypic heterogeneity in cell populations through its parasexual cycle (22, 23) by reassorting chromosomes into new allelic combinations, which has been proposed to facilitate rapid adaptation (15). Others have demonstrated that decreased heterozygosity correlates with decreased in pathogen fitness and virulence (29, 39, 51). In our study, we measured genome size changes but have not yet characterized the allelic composition in our host-derived isolates. Thus, it is possible that after undergoing massive host-associated genome size reductions, tetraploid derivatives contained fewer heterozygous chromosomes. When we consider the large genomic but small phenotypic changes in tetraploids, coupled with the small genomic but large phenotypic changes in diploids, we propose that specific allelic combinations and potential *de novo* mutations foster virulence changes.

## MATERIALS AND METHODS

### Strains and maintenance.

We used six C. albicans strains for this study that varied in their ploidy and their genetic background (Table 1). Each strain was initially struck out from glycerol stocks stored at −80°C onto a YPD (yeast extract-peptone-dextrose [1% yeast extract, 2% Bacto peptone, 2% glucose, 1.5% agar, 0.004% adenine, 0.008% uridine]) plate. After 48 h at 30°C, a single colony was arbitrarily chosen as the “parental strain.”

To construct diploid clinical strains heterozygous for the *GAL1* locus, we transformed one copy of the *GAL1* open reading frame in FH1 and PN2 with the dominant drug-resistant *NAT* gene marker by lithium acetate transformation. *NAT* was amplified from plasmid pMG2120 (52) by PCR (step 1, 94°C for 5 min; step 2, 94°C for 30 s; step 3, 55°C for 45 s; step 4, 72°C for 4 min; step 5, repeat steps 2 through 4 29 times; step 6, 72°C for 10 min) using primers oMH112 and oMH113 listed in Table S1 in the supplemental material). Transformants were selected on YPD containing 50 μg/ml nourseothricin. PCR (step 1, 94°C for 3 min; step 2, 94°C for 30 s; step 3, 55°C for 30 s; step 4, 68°C for 2 min; step 5, repeat steps 2 through 4 35 times; step 6, 68°C for 10 min) was performed to verify that *NAT* was properly integrated using primers oMH106, oMH5, and oMH104 (Table S1). All strains were stored at −80°C and maintained on YPD at 30°C.

10.1128/mSphere.00433-20.3 TABLE S1 Oligonucleotides used in this study. Download Table S1, PDF file, 0.02 MB. Copyright © 2020 Smith and Hickman. 2020 Smith and Hickman This content is distributed under the terms of the Creative Commons Attribution 4.0 International license.

10.1128/mSphere.00433-20.4 TABLE S2 Diploid LOH frequency. Mean LOH frequency, standard deviation (SD), and number of biological replicates (*n*) are indicated for each treatment. Differences between treatments were tested in all pairwise combinations using the Mann-Whitney U test, and significance is indicated (ns, not significant; *, *P* < 0.05; **, *P* > 0.01; ***, *P* > 0.001; ****, *P* > 0.0001). Download Table S2, PDF file, 0.01 MB. Copyright © 2020 Smith and Hickman. 2020 Smith and Hickman This content is distributed under the terms of the Creative Commons Attribution 4.0 International license.

10.1128/mSphere.00433-20.5 TABLE S3 Characteristics of host-associated isolates. For each genetic background and ploidy, the genome size (measured as mean G_1_ FITC signal), growth rate, and infected host fecundity values are indicated. Download Table S3, PDF file, 0.02 MB. Copyright © 2020 Smith and Hickman. 2020 Smith and Hickman This content is distributed under the terms of the Creative Commons Attribution 4.0 International license.

C. elegans N2 Bristol (wild type) was used for fecundity and host-associated genome stability assays. C. elegans populations were maintained on plates containing nematode growth medium (NGM) with Escherichia coli (OP50) for a food source. C. elegans was transferred to a new plate containing freshly seeded E. coli every 3 to 4 days. For genome stability assays, treatment plates were seeded with both C. albicans and E. coli and supplemented with 0.2 g/liter streptomycin to inhibit overgrowth of E. coli. For fecundity and genome stability assays, NGM was supplemented with 0.08 g/liter of uridine and 0.08 g/liter of histidine to facilitate growth of auxotrophic C. albicans strains.

### Seeding NGM plates for genome stability assays.

Single colonies of C. albicans were inoculated into 3 ml of YPD and incubated at 30°C overnight. C. albicans cultures were diluted to a final volume of an optical density at 600 nm (OD_600_) of 3.0 per ml in double-distilled water (ddH_2_O). Additionally, E. coli was inoculated into 50 ml of LB and incubated at 30°C for 24 to 48 h. Subsequently, E. coli was pelleted and washed twice with 1 ml of ddH_2_O. The washed pellet was then weighed and diluted to a final density of 200 mg/ml. *In vitro* treatment plates contained 250 μl of diluted C. albicans plated and spread onto an NGM plus streptomycin agar plate and incubated overnight at 30°C. *In vivo* treatment plates had 6.25 μl of C. albicans and 31.25 μl of E. coli and were brought to a final volume of 250 μl with ddH_2_O. This mixture was plated and spread onto an NGM plus streptomycin agar plate and incubated overnight at 30°C.

### Seeding NGM plates for fecundity assays.

Seeding NGM plates and synchronizing C. elegans populations for fecundity assays were performed as previously described (36). Briefly, C. albicans cultures were inoculated into 3 ml of YPD and incubated at 30°C overnight. C. albicans cultures were diluted to a final volume of 3.0 OD_600_ per ml. Additionally, E. coli was inoculated into 50 ml of LB and incubated at 30°C for 24 to 48 h. Subsequently, E. coli was pelleted and washed twice with 1 ml of ddH_2_O. The washed pellet was then weighed and diluted to a final density of 200 mg/ml. Day 0 uninfected treatment plates contained 6.25 μl of E. coli and were brought to a final volume of 50 μl with ddH_2_O. Day 0 C. albicans treatment plates had 1.25 μl of C. albicans and 6.25 μl of E. coli and were brought to a final volume of 50 μl. The entire 50 μl was spotted onto the center of a 35-mm-diameter NGM plus streptomycin agar plate, followed by incubation at room temperature overnight before the addition of eggs or transferring nematodes. For days 2 to 7 of the experiment, C. albicans treatment plates contained 0.25 μl of C. albicans, 1.25 μl of E. coli, and 8.5 μl of ddH_2_O. For uninfected treatments, 1.25 μl of E. coli was mixed with 8.75 μl of ddH_2_O. The entire 10 μl was spotted onto a 35-mm-diameter NGM plus streptomycin agar plate, followed by incubation at room temperature overnight before the transfer of nematodes.

### Egg preparation and synchronization for genome stability and fecundity assays.

To synchronize C. elegans populations, nematodes and eggs were washed off the plate with an M9 buffer, transferred to a 15-ml conical tube, and pelleted at 1,200 rpm for 2 min. The pellet was resuspended in a 25% bleach solution, inverted for 2 min, and subsequently centrifuged for 2 min at 1,200 rpm. The pellet was washed twice with 3 ml of ddH_2_O and resuspended in 1 ml of ddH_2_O. To determine the concentration of eggs, 10 μl was pipetted onto a concave slide, the eggs were counted under a microscope, and the egg suspension was diluted with M9 to a concentration of ∼100 eggs per 100 μl.

### Host-associated yeast extractions.

C. elegans worms colonized with C. albicans were washed off the plate with 3 ml of M9 worm buffer. This suspension was centrifuged for 2 min at 2,000 rpm to pellet the worms. The supernatant was removed, and 1 ml of 3% bleach was added, transferred to a microcentrifuge tube, and incubated for 3 min. The worm suspension was centrifuged for 1 min at 12,000 rpm. The supernatant was removed, washed with 1 ml of M9, and centrifuged for 1 min at 12,000 rpm. The wash was repeated two more times to ensure that all bleach was removed. One-hundred-microliter aliquots of nematode suspension were transferred to 0.6-ml clear microtubes for manual disruption with a motorized pestle. After 1 min of manual disruption, the worm intestine solution was then diluted accordingly with an M9 buffer and plated on YPD plus 0.034 mg/liter chloramphenicol to select prevent any bacterial colonies from arising.

### *GAL1* loss-of-heterozygosity assay. (i) *In vitro*.

Single colonies of C. albicans were inoculated in 3 ml YPD, grown overnight at 30°C, and subsequently diluted to an OD of 3 in ddH_2_O. Two hundred fifty microliters was plated, spread onto NGM plus streptomycin plates, incubated overnight at 30°C, and transferred to 20°C for 4 days. On day 4, yeast cells were washed off with ddH_2_O, harvested by centrifugation, washed once with ddH_2_O, resuspended in 1 ml of ddH_2_O, and serially diluted for single-colony growth. To determine the total cell viability, 100 μl of a 10^−6^ dilution was plated onto YPD and grown for 48 h at 30°C. To identify cells that lost *GAL1*, 100 μl of 10^−2^ and 10^−3^ dilutions was plated onto 2-deoxygalactose (2-DOG; 0.17% yeast nitrogen base without amino acids, 0.5% ammonium sulfate, 0.0004% uridine, 0.0004% histidine, 0.1% 2-deoxygalactose, 3% glycerol), and CFU were counted following 72 h of incubation at 30°C.

### (ii) *In vivo*.

The *in vivo* approach was very similar to the *in vitro* LOH assay described above, with the following changes. A population of ∼100 nematodes was plated on each treatment plate containing both C. albicans and E. coli. On day 4, yeasts were extracted as described above. Dilutions of 10^−1^ and 10^−2^ were plated on YPD plus chloramphenicol to enumerate total growth, and undiluted cells were plated on 2-DOG to select for the loss of *GAL1.* Three technical replicates were used for each C. albicans strain for both *in vitro* and *in vivo* experiments. At least three biological replicates were used for each genome stability assay.

### *In vitro* passaging.

Serial passaging experiments were performed as previously described (23). Briefly, we inoculated 36 single-colony isolates for each C. albicans strain into 500 μl liquid YPD in 96-deep-well culture blocks and incubated them at 30°C with shaking. Every 24 h, 5 μl of culture was diluted in 495 ml fresh YPD (1:100 dilution) and incubated at 30°C with shaking. On days 4, 7, 14, and 28, cultures were simultaneously prepared for flow cytometry or for long-term storage in 50% glycerol at −80°C. Glycerol stocks were also prepared on days 10, 17, 21, and 24.

### Flow cytometry for genome size determination.

Single yeast colonies extracted from C. elegans were inoculated in YPD and incubated overnight at 30°C. Samples were subcultured into wells with 495 μl of fresh YPD and grown at 30°C for an additional 6 h. Cells were subsequently collected by centrifugation (1,000 rpm, 5 min), and the supernatant was removed and resuspended in 50:50 TE (50 mM Tris, pH 8, and 50 mM EDTA). Cells were fixed by adding 180 μl of 95% ethanol and stored overnight at 4°C. Following fixation, cells were collected by centrifugation, washed with 50:50 TE, and treated with 50 μl of RNase A (1 mg/ml) for 1 h at 37°C with shaking. Following RNase treatment, cells were collected by centrifugation, RNase solution was removed, and cells were resuspended with 50 μl proteinase K (5 mg/ml) and incubated for 30 min at 37°C. Following proteinase K treatment, cells were collected by centrifugation, washed once with 50:50 TE, resuspended in 50 μl Sybr green (1:50 dilution with 50:50 TE; Lonza, catalog no. 12001-798, 10,000×), and incubated overnight at 4°C. Following Sybr green straining, cells were collected by centrifugation, Sybr green was removed, and cells were resuspended in 50 μl 50:50 TE. Samples were sonicated to disrupt any cell clumping and subsequently run on an LSRII flow cytometer. To calibrate the LSRII and serve as internal controls, the reference diploid (SC5314) and tetraploid strains were used.

Flow cytometry data were analyzed using FlowJo, by plotting the fluorescein isothiocyanate A (FITC-A) signal against the cell count. Two peaks were observed, the first representing the G_1_ mean and the second peak representing the G_2_ mean, which has double the genome content of the G_1_ peak and therefore twice the fluorescence. Genome size values were calculated using the G_1_ mean and compared to standard diploid and tetraploid control strains.

### Host fecundity assays.

Approximately 50 host eggs were added to each control and treatment plate. After 48 h of growth at 20°C, a single L4 nematode (× 10 per treatment) was randomly selected and transferred to an uninfected or C. albicans treatment plate and incubated at 20°C. Each nematode was transferred to a new plate every 24 h for 5 consecutive days. Any eggs laid for each 24-h interval were incubated for 24 h at 20°C, and the number of viable progenies produced per worm was enumerated.

### Growth rate assays.

Twelve random colonies for each parental strain and 7 colonies for each host-associated isolate were grown overnight in 450 μl of YPD in a 96-well block at 30°C with shaking. Cultures were diluted 10-fold with ddH_2_O. Fifteen microliters of diluted culture was inoculated into 135 μl of YPD in a sterile round-bottom 96-well plate and placed on the BioTek ELx808 absorbance microplate reader. Optical density (OD) was measured every 15 min for 24 h at 30°C with shaking. Growth rate was determined using a custom R script that calculates the maximal growth rate in each well as the spline with the highest slope from a loess fit through log-transformed optical density data that reflect the rate of cell doubling (developed by Richard Fitzjohn, as in reference 53).

### Statistical analysis.

Statistical analysis was performed using GraphPad Prism 8 software. Data sets were tested for normality using the D’Agostino and Pearson omnibus normality test. Student’s *t* tests were used to test for differences between LOH frequencies for host and no-host treatments. Deviations from initial genome size were determined by first pooling the day 0 data for all the diploid or tetraploid strains to calculate the mean and standard deviation (SD). Replicate lines ±1 SD from the day 0 mean were considered deviations. *In vivo* genome size deviations were calculated similarly; however, the mean and standard deviations were calculated for each strain rather than being pooled. Unpaired, Mann-Whitney U-tests were used to test for differences in growth rate and host fecundity between host-associated isolates and their respective parental strains.

### Data availability.

All relevant data are posted on the Dryad Digital Repository at the following URL: https://datadryad.org/stash/share/IVyqvDjX_Ly_IaCiyuL1xa813uBuvlLLMJ5Gg4-fPQw.
