# Supplementary figures and images for: Host-Induced Genome Instability Rapidly Generates Phenotypic Variation across Candida albicans Strains and Ploidy States
Source: mSphere. 2020 Jun 3;5(3):e00433-20. doi: 10.1128/mSphere.00433-20 (PMC7273350; doi:10.1128/mSphere.00433-20)

A.

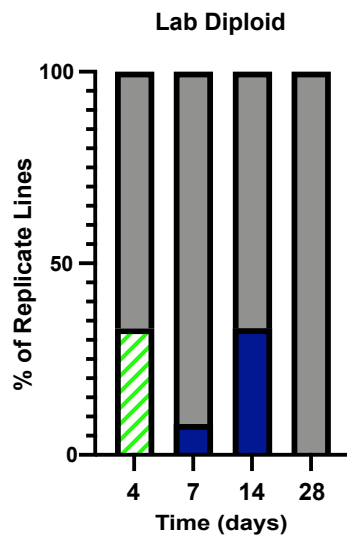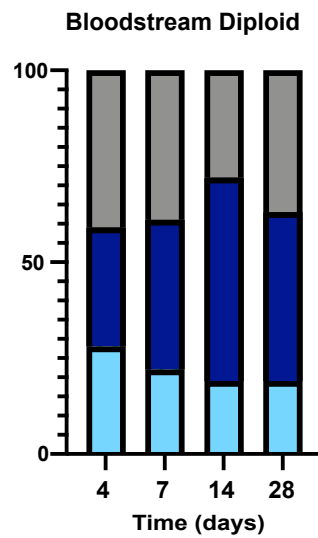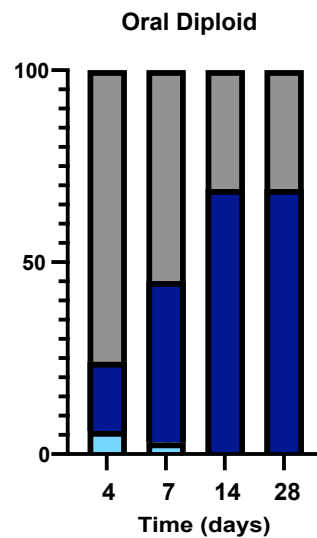

B.

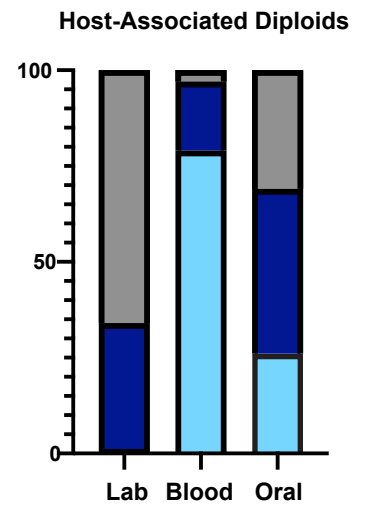

No change
  Mixed
  Losses
  Gains

C.

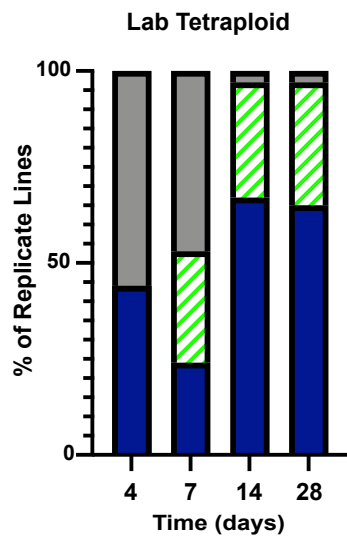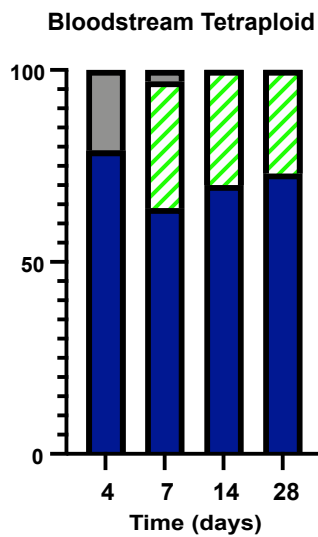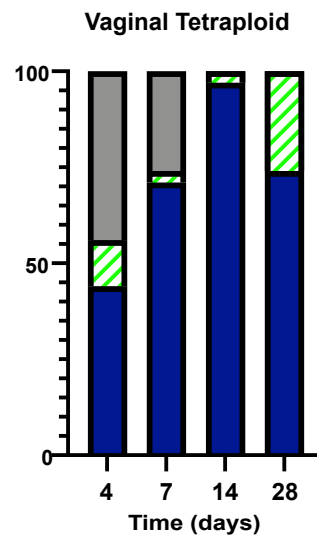

D.

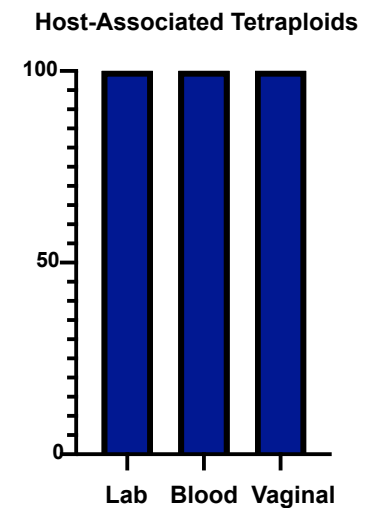

No change
  Mixed
  Losses
  Gains

Supplement: FIG S1 [file mSphere.00433-20-sf001.pdf]
