# Supplementary material for: Host-Induced Genome Instability Rapidly Generates Phenotypic Variation across Candida albicans Strains and Ploidy States
Source: mSphere. 2020 Jun 3;5(3):e00433-20. doi: 10.1128/mSphere.00433-20 (PMC7273350; doi:10.1128/mSphere.00433-20)
Supplement: FIG S2 [file mSphere.00433-20-sf002.pdf]

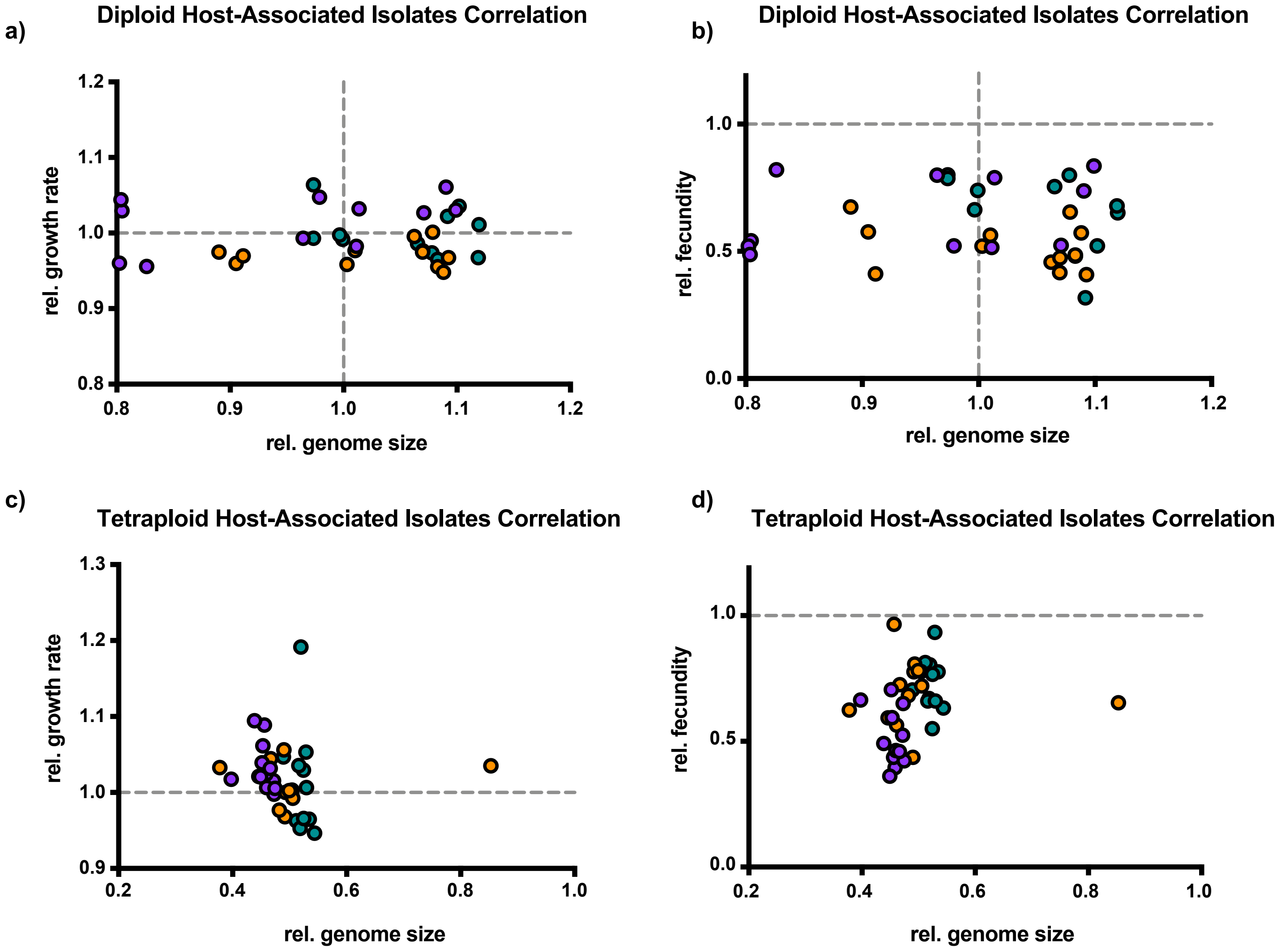
a)
Diploid Host-Associated Isolates Correlation
rel. growth rate
1.2
1.1
1.0
0.9
0.8
0.8
0.9
1.0
1.1
1.2
rel. genome size
b)
Diploid Host-Associated Isolates Correlation
rel. fecundity
1.0
0.5
0.0
0.8
0.9
1.0
1.1
1.2
rel. genome size
c)
Tetraploid Host-Associated Isolates Correlation
rel. growth rate
1.3
1.2
1.1
1.0
0.9
0.2
0.4
0.6
0.8
1.0
rel. genome size
d)
Tetraploid Host-Associated Isolates Correlation
rel. fecundity
1.0
0.5
0.0
0.2
0.4
0.6
0.8
1.0
rel. genome size
