# Supplementary material for: Host-Induced Genome Instability Rapidly Generates Phenotypic Variation across Candida albicans Strains and Ploidy States
Source: mSphere. 2020 Jun 3;5(3):e00433-20. doi: 10.1128/mSphere.00433-20 (PMC7273350; doi:10.1128/mSphere.00433-20)
Supplement: TABLE S1 [file mSphere.00433-20-st001.pdf]

Primer name	Sequence	Direction	Purpose
oMH112	5'-CGGTTTCCGGGGTTGGTGTTTAGTTTTCGTTTTTCGTTTTTTTTGGAAAGAATGTTTTAGCTCATTGACTGGATGGCGGCGTTAGTATCG-3'	Forward	Tagging primer to replace *GAL1* with *NAT* using pMH10
oMH113	5'-ATTTGTTTTGACCATATGGTAGTTGCGATATTCGTCGCCCTATCTATTTTTGCAACAGACTAATACCGTAAAACGACGGCCAGTGAATTC-3'	Reverse	Tagging primer to replace *GAL1* with *NAT* using pMH10
oMH104	5'-CTGGTTGGGGTGGTTCAATTG-3'	Forward	Verification of *GAL1*
oMH106	5'-GGTAGTTGCGATATTCGTCGC-3'	Reverse	Verification of *GAL1/NAT*
oMH5	5'-GGTGGATCAACTGGAACTTCTC-3'	Forward	Verification of *NAT*
