# Supplementary material for: Host-Induced Genome Instability Rapidly Generates Phenotypic Variation across Candida albicans Strains and Ploidy States
Source: mSphere. 2020 Jun 3;5(3):e00433-20. doi: 10.1128/mSphere.00433-20 (PMC7273350; doi:10.1128/mSphere.00433-20)
Supplement: TABLE S2 [file mSphere.00433-20-st002.pdf]

Treatment		*in vitro*			Host associated		
		**Laboratory**	**Bloodstream**	**Oral**	**Laboratory**	**Bloodstream**	**Oral**
**Mean LOH** ±*SD*		0.00013<br>*0.000079*<br>n=9	0.0085<br>0.0043<br>n=9	0.0002<br>0.000095<br>n=12	0.0015<br>0.001<br>n=8	0.022<br>0.025<br>n=12	0.0019<br>*0.0023*<br>n=9
in vitro	**Laboratory**						
	**Bloodstream**	****					
	**Oral**	ns	****				
Host associated	**Laboratory**	****	***	****			
	**Bloodstream**	****	ns	****	***		
	**Oral**	****	**	****	ns	***	
