# Supplementary material for: Host-Induced Genome Instability Rapidly Generates Phenotypic Variation across Candida albicans Strains and Ploidy States
Source: mSphere. 2020 Jun 3;5(3):e00433-20. doi: 10.1128/mSphere.00433-20 (PMC7273350; doi:10.1128/mSphere.00433-20)
Supplement: TABLE S3 [file mSphere.00433-20-st003.pdf]

| Group | | Strain | Diploids | | | Tetraploids | | |
|---|---|---|---|---|---|---|---|---|
| | | | Genome size | Growth Rate ±1SD (n) | Rel. Fecundity ±1SD (n) | Genome size | Growth Rate ±1SD (n) | Rel. Fecundity ±1SD (n) |
| Laboratory | | Parent | 45,915 | 0.47±0.02 (12) | .99±.12 (27) | 96,000 | 0.42 ± .02 (12) | .56 ± .10 (10) |
| Laboratory | Host-associated derivatives | 1 | 45,882 | 0.47±0.01 (3) | .74±.20 (8) | 49,911 | 0.50 ± .005 (3) | .80 ± .07 (7) |
| Laboratory | Host-associated derivatives | 2 | 48,906 | 0.46±0.01(3) | .75±.14 (9) | 49,128 | 0.48 ± 0.02 (3) | .81 ± .19 (8) |
| Laboratory | Host-associated derivatives | 3 | 45,755 | 0.46±0.01(3) | .66±.09 (8) | 51,312 | 0.47 ± 0.03 (3) | .78 ±.10 (8) |
| Laboratory | Host-associated derivatives | 4 | 49,496 | 0.45±0.01 (3) | .80±.15 (7) | 48,403 | 0.47 ± 0.02 (3) | .78 ± .14 (8) |
| Laboratory | Host-associated derivatives | 5 | 44,699 | 0.45±0.01 (3) | .80±.08 (10) | 46,948 | 0.49 ± 0.02 (3) | .70 ± 22 (8) |
| Laboratory | Host-associated derivatives | 6 | 50,593 | 0.46±0.01 (3) | .52±.16 (7) | 49,753 | 0.47 ± 0.02 (3) | .67 ± .17 (5) |
| Laboratory | Host-associated derivatives | 7 | 49,721 | 0.45±0.01 (3) | .48±.11 (8) | 50,343 | 0.48 ± 0.02 (3) | .55 ± .06 (6) |
| Laboratory | Host-associated derivatives | 8 | 51,403 | 0.45±0.02 (3) | .65±.11 (8) | 52,191 | 0.46 ± 0.04 (3) | .63 ± .11 (8) |
| Laboratory | Host-associated derivatives | 9 | 56,532 | 0.46±0.003 (3) | .48±.10 (5) | 49,562 | 0.47 ± 0.03 (3) | .66± .19 (9) |
| Laboratory | Host-associated derivatives | 10 | 50,120 | 0.47±0.002 (3) | .32±.04 (3) | 50,347 | 0.46 ± 0.03 (3) | .77 ± .14 (7) |
| Laboratory | Host-associated derivatives | 11 | 51,364 | 0.45±0.01 (3) | .68±.20 (7) | 50,851 | 0.46 ± 0.01 (3) | .66± .16 (5) |
| Laboratory | Host-associated derivatives | 12 | 44,692 | 0.48±0.02 (3) | .79±.24 (6) | 50,756 | 0.48 ± 0.01 (3) | .93 ± .17 (7) |
| Bloodstream | | Parent | 49,040 | .47 ± .02 (12) | .71± .10 (15) | 102,918 | .43 ± 0.01 (12) | .53 ± .12 ( 6) |
| Bloodstream | Host-associated derivatives | 1 | 52,094 | 0.47±0.05 (7) | .46±.04 (5) | 45,892 | 0.44 ± 0.03 (7) | .59 ± .09 (8) |
| Bloodstream | Host-associated derivatives | 2 | 52,470 | 0.46±0.03 (7) | .47±.09 (5) | 47,018 | 0.44 ± 0.01 (7) | .96 ± .16 (10) |
| Bloodstream | Host-associated derivatives | 3 | 44,389 | 0.45±0.03 (7) | .57±.09 (5) | 50,442 | 0.45 ± 0.01 (7) | .43 ± .12 (7) |
| Bloodstream | Host-associated derivatives | 4 | 53,108 | 0.45±0.02 (7) | .49±.03 (6) | 47,452 | 0.44 ± 0.01 (7) | .57 ± .14 (8) |
| Bloodstream | Host-associated derivatives | 5 | 44,699 | 0.46±0.04 (7) | .41±.08 (8) | 50,599 | 0.42 ± 0.02 (7) | .77± .22 (5) |
| Bloodstream | Host-associated derivatives | 6 | 52,456 | 0.46±0.02 (7) | .42±.06 (5) | 50,743 | 0.43 ± 0.02 (7) | .81 ± .30 (4) |
| Bloodstream | Host-associated derivatives | 7 | 53,578 | 0.46±0.03 (7) | .41±.11 (7) | 52,037 | 0.43 ± 0.02 (7) | .72 ± .16 (4) |
| Bloodstream | Host-associated derivatives | 8 | 53,358 | 0.45±0.03 (7) | .57±.11 (8) | 48,062 | 0.45 ± 0.03 (7) | .73 ± .18 (6) |
| Bloodstream | Host-associated derivatives | 9 | 43,653 | 0.46±0.02 (7) | .67± .00 (2) | 51,368 | 0.43 ± 0.02 (7) | .78 ± .15 (5) |
| Bloodstream | Host-associated derivatives | 10 | 49,532 | 0.46±0.01 (7) | .56±.09 (6) | 49,621 | 0.42 ± 0.01 (7) | .68 ± .11 (5) |
| Bloodstream | Host-associated derivatives | 11 | 49,182 | 0.45±0.01 (7) | .52±.13 (7) | 38,843 | 0.44 ± 0.03 (7) | .62 ± .11 (10) |
| Bloodstream | Host-associated derivatives | 12 | 52,892 | 0.47±0.02 (7) | .65±.14(9) | 87,795 | 0.44 ± 0.01 (7) | .65 ± .13 (6) |
| Oral/Vaginal | | Parent | 50,680 | 0.38±0.03 (12) | .68±.14 (18) | 98,243 | .45 ± 0.03 (12) | .48±.08 (7) |
| Oral/Vaginal | Host-associated derivatives | 1 | 54,272 | 0.42±0.01 (7) | .52±.13 (5) | 46,350 | 0.46 ± 0.02 (7) | .52 ± .09 (4) |
| Oral/Vaginal | Host-associated derivatives | 2 | 51,373 | 0.43±0.01 (7) | .97±.16 (6) | 45,041 | 0.47 ± 0.02 (7) | .39 ± .01 (2) |
| Oral/Vaginal | Host-associated derivatives | 3 | 49,609 | 0.43±0.01 (7) | .52±.07 (7) | 44,761 | 0.49 ± 0.02 (7) | .44 ± .08 (5) |
| Oral/Vaginal | Host-associated derivatives | 4 | 40,777 | 0.43±0.03 (7) | .54±.17 (7) | 43,069 | 0.49 ± 0.05 (7) | .49 ± .12 (5) |
| Oral/Vaginal | Host-associated derivatives | 5 | 55,695 | 0.43±0.02 (7) | .84±.08 (5) | 46,420 | 0.45 ± 0.02 (7) | .65 ± .09 (5) |
| Oral/Vaginal | Host-associated derivatives | 6 | 48,867 | 0.41±0.01 (7) | .80±.15 (9) | 44,373 | 0.47 ± 0.03 (7) | .71 ± .09 (5) |
| Oral/Vaginal | Host-associated derivatives | 7 | 55,257 | 0.44±0.01 (7) | .74±.17 (6) | 44,528 | 0.48 ± 0.02 (7) | .59 ± .10 (6) |
| Oral/Vaginal | Host-associated derivatives | 8 | 41,878 | 0.40±0.02 (7) | .82±.05 (8) | 39,051 | 0.46 ± 0..01 (7) | .66 ± .10 (4) |
| Oral/Vaginal | Host-associated derivatives | 9 | 39,722 | 0.42±0.04 (7) | .49±.10 (9) | 45,178 | 0.46 ± .0.02 (7) | .46 ± .15 (6) |
| Oral/Vaginal | Host-associated derivatives | 10 | 51,250 | 0.41±0.02 (7) | .52±.11 (10) | 44,115 | 0.46 ± 0.03 (7) | .36 ± .13 (4) |
| Oral/Vaginal | Host-associated derivatives | 11 | 40,667 | 0.40 ± .01 (7) | .52±.10 (10) | 46,617 | 0.46 ± 0.02 (7) | .42± .07 (3) |
| Oral/Vaginal | Host-associated derivatives | 12 | 40,727 | 0.43 ± .01 (7) | .49 ± .08(10) | 45,786 | 0.47 ± 0.03 (7) | .46± .03 (4) |
